# Treatment of Pneumonitis

**Published:** 1874-02-15

**Authors:** A. Hermann

**Affiliations:** Pesth


### Translations.

### TREATMENT OF PNEUMONITIS.

By Dr. A. Hermann, of Pesth.

*Translated for* The Examiner, *from the Allgemeine Wiener Med. Zeitung, by* H. Gradle.

Induced by the pamphlet of Juergensen, on the treatment of pneumonitis,* the author gives us, in this article, the result of his experience in that disease, in a form valuable on account of its accuracy and minuteness. Between July 1st, 1866, and Dec. 31st, 1872, one hundred and eighty-six cases of pneumonitis were treated in the hospital at Pesth, a number perhaps not very large, but still almost equalling the basis of

* Clinical Lectures, edited by R. Volkmann, Grundsaetze fuer die Behandlung der Croupoesen Pneumonie von Th. Juergensen, 1872.

Juergensen's conclusions. To avoid any misinterpretation, the author plainly states, that by the term pneumonitis, he refers to *the acute affection, complicated with fever, in which a fibrinous exudation takes place into the alveoli of the lungs, and in which the fever has a definite type*, i.e., *running its course in a more or less distinct cycle*.

This definite cycle can, however, be observed only when the disease affects a previously healthy individual, and is uncomplicated, since its natural history would be adulterated, so to speak, by any intercurrent trouble. Neither ought secondary *croupous* inflamations of the lung be studied in this connection, as the clinical appearances do not present such well-marked limits; besides, the impression left on the system by the preceding illness, or even the lingering of the morbid process, materially affects the character of the supervening pulmonary disease, and leaves us in doubt as to the origin of the gravity of symptoms, whether caused by the primary or secondary malady. In fact, not unfrequently is it difficult to establish the very presence of the disease turning the course of typhus, scarlatina, variola, etc. The thermometer, so deserving of just reliance, though indicating a variation from the normal history of these disorders, seldom, if ever, enables the observer to render a positive diagnosis. This circumstance the author holds responsible for the discrepancy in the statements of different writers. For this reason, though acknowledging that phthisical and malarial patients may present the distinct cycle, all impure cases (23) have been excluded from the analysis, leaving for consideration one hundred and sixty-three typical ones, divided among one hundred and thirty-two males and thirty-one females.

The twenty-three complicated cases in whom the cycle was also not ill-defined can be tabulated, as regards the concurrent affection: as

| | | | |
|---|---|---|---|
| Pulmonary phthisis | 9 | Sarcoma of the submaxillary bone, | 1 |
| Pneumorrhagia, | 1 | Syphilis, | 1 |
| Erysipelas, | 1 | Intermittent fever, | 1 |
| Pericarditis, | 1 | Pleurisy, | 1 |
| Chronic cystitis, | 1 | Relapsing fever, with metastatic parotitis, | 1 |
| Variola, | 1 | | — |
| Bright's disease, | 1 | Total, | 22 |
| Typhus, | 1 | (One case omitted by the author.) | |
| Icterus (bilious pneumonia), | 1 | | |

Any practitioner accustomed to making careful records of his cases, soon recognizes that the distinct cycle, previously defined as essential to pneumonitis, is generally very manifest; thus, in forty patients observed, in whom the frequency of the pulse and the temperature of the axilla were twice daily ascertained, viz.: 8 A.M. and 5 P.M., defervescence took place, when counting the beginning of the disease, from the time of the first rigor, or the day when the patient sought the bed:

| | | | |
|---|---|---|---|
| On the 3d day, | 1 time | On the 9th day, | 4 times |
| " 4th day, | 1 " | " 10th day, | 1 time |
| " 5th day, | 5 times | " 11th day, | 2 times |
| " 6th day, | 6 " | " 12th day, | 1 time |
| " 7th day, | 12 " | " 16th day, | 1 " |
| " 8th day, | 5 " | " 21st day, | 1 " |

The termination of these cases was, in all, a happy one; and though the entire number is somewhat limited, it suffices to demonstrate the regularity of duration reported by other observers. Defervescence occurs most frequently on the seventh day; and within the first seven days, no less than twenty-five patients had convalesced. The eighth day brought relief to five persons, and the ninth to four; thus the majority of cases enter on convalescence within nine days, while but few endure the affection for a longer period. The manner of defervescence separates the cases into two groups; in the first of which the apyrexia is rapidly produced, and the interval between a morning and evening visit decides as regards the result.

The return of the animal heat to the normal standard is accompanied by a corresponding diminution of the frequency of the pulse, when a favorable issue is apparent; and this rule seems so constant, that a simultaneous *increase* in the rapidity of the cardiac contractions justifies a grave prognosis.

Frequently, though by no means always, was the approach of a happy termination preceded by copious perspiration, especially on the face and trunk. As representatives of this group the following cases may be cited:

CASE I.—E. L., laborer, aged twenty, entered hospital January 5, 1872, with inflammation of the right lung; discharged January 19, 1872.

| | |
|---|---|
| 3d day, evening, T 102.9<br>P 108. | 5th day, evening, T 103.1<br>P 104. |
| 4th day, morning, T 102.9<br>P 104. | 6th day, morning, T 103.1<br>P 108. |
| 4th day, evening, T 103.3<br>P 100. | 6th day, evening, T 104.7<br>P 108. |
| 5th day, morning, T 102.9<br>P 100. | 7th day, morning, T 97.8<br>P 64. |

CASE II.—H. C., aged sixteen, entered November 8, 1871, with pneumonitis of the left side; discharged December 1, 1871.

| | |
|---|---|
| 3d day, evening, T 102.2<br>P 112. | 8th day, morning, T 103.2<br>P 112. |
| 4th day, morning, T 102.5<br>P 120. | 8th day, evening, T 103.6<br>P 120. |
| 4th day, evening, T 103.3<br>P 116. | 9th day, morning, T 103.3<br>P 120. |
| 5th day, morning, T 104.3<br>P 120. | 9th day, evening, T 102.4<br>P 120. |
| 5rh day, evening, T 104.7<br>P 120. | 10th day, morning, T 103.1<br>P 120. |
| 6th day, morning, T 102.9<br>P 112. | 10th day, evening, T 104.1<br>P 128. |
| 6th day, evening, T 104.5<br>P 132. | 11th day, morning, T 102.7<br>P 120. |
| 7th day, morning, T 104.1<br>P 132. | 11th day, evening, T 104.0<br>P 120. |
| 7th day, evening, T 104.0<br>P 120. | 12th day, morning, T 98.2<br>P 88. |

CASE III.—A. S., aged forty-one, entered May 16, 1872, with pneumonitis of right side; died May 20, 1872.

| | |
|---|---|
| 3d day, evening, T 101.4<br>P 112. | 6th day, morning, T 98.9<br>P 116. |
| 4th day, morning, T 102.3<br>P 116. | 6th day, evening, T 98.6<br>P 124, almost imperceptible. |
| 4th day, evening, T 102.9<br>P 112. | 7th day, 8 A.M., T 98.9<br>P 116, almost imperceptible. |
| 5th day, morning, T 98.9<br>P 120, small. | 7th day, 10 A.M., death. |
| 5th day, evening, T 99.6<br>P 124. | |

A second class is constituted by cases in which defervescence is a gradual process, an interval of at least thirty-six to forty hours elapsing before the decline of temperature has reached the normal standard. As samples of this class of cases, the following tables may be cited:

CASE I.—H. B., aged twenty-five, April 7–27, 1872; inflammation of right lung.

| | |
|---|---|
| 2d day, evening, T 102.9<br>P 108. | 5th day, evening, T 103,6<br>P 104. |
| 3d day, morning, T 103.2<br>P 104. | 6th day, morning. T 103.1<br>P 100. |
| 3d day, evening, T 103.1<br>P 96. | 6th day, evening, T 101.5<br>P 108. |
| 4th day, morning, T 102.9<br>P 100. | 7th day, morning, T 99.6<br>P 96. |
| 4th day, evening, T 104.0<br>P 108. | 7th day, evening, T 100.7<br>P 84. |
| 5th day, morning, T 102.5<br>P 190. | 8th day, morning, T 99.3<br>P 72. |

CASE II.—M. K., aged twenty-eight, April 10–30, 1872; disease of right lung.

| | |
|---|---|
| 3d day, evening, T 103.2<br>P 112. | 5th day, evening, T 101.6<br>P 96 |
| 4th day, morning, T 102.9<br>P 108. | 6th day, morning, T 100.4<br>P 88. |
| 4th day, evening, T 102.9<br>P 104. | 6th day, evening, T 100.2<br>P 84. |
| 5th day, morning, T 101.8<br>P 100. | 7th day, morning, T 98.9<br>P 76. |

Though deviations from these two types do occur, they only serve to establish the rule; but other signs and symptoms, as abnormal appearances revealed by auscultation and percussion, continue to persist for some days, only when the febrile condition was of but three days' duration.

The doctrine, however, of Juergensen, that symptoms, resembling in their clinical aspect the commencement of pneumonitis, may have disappeared completely in twenty-four to thirty-six hours, the author hesitates to accept, not daring to give a positive diagnosis, unless confimed by the subsequent history, since, under such vague circumstances, therapeutic measures could not be fairly criticised.

A correct estimate of the efficacy of any therapeutic measures can, rationally, be only formed by a comparison of results with the undisturbed course of the disease in question; and only he who has dared to study the true "natural history" of an affection, can legitimately boast of the success of his treatment. A rude comparison of cases under different remedial influences, is by no means an unequivocal basis for conclusions such as Juergensen (loc. cit.) has arrived at, since a large variety of circumstances may alter the conditions attending the cases.

As before stated, the author analyzed one hundred and sixty-three cases of genuine pneumonitis, of which twenty-five succumbed to the disease, thus making the mortality 15.34 per cent. Of twenty-three cases, previously cited as being complicated, there were five deaths, increasing the fatal termination of one hundred and eighty-six to thirty, or 16.13 per cent. Apart from treatment, the author now enters into statistics of facts. Of one hundred and sixty-three genuine cases, there were:

| | | | | | | | |
|---|---|---|---|---|---|---|---|
| Inflam'n of right lung, | 81, | with | 12 | deaths, | or | 14.81 | p.c. |
| " " left " | 50, | " | 3 | " | " | 6.00 | " |
| " " both " | 20, | " | 5 | " | " | 25.00 | " |
| Not known which side affected, | 12, | " | 5 | " | " | 41.67 | " |

This compilation proves disease of the left side to present a more favorable prognosis than the affection of the right, and especially the involvement of both lungs. Similar results are also obtained from the analyses of other writers. It is evident that the extent of infiltration must determine the gravity of the affection. The greater size, therefore, of the right lung, would increase the danger, if infiltrated with œdematous exudation; which is, besides, more likely to occur on that side, from the relatively greater length of the right pulmonary vein, augmenting the resistance to the return of the arterialized blood to the heart. As regards age, one hundred and sixty cases of uncomplicated pneumonitis, whose age was ascertained, showed the following relations:

| | | | | | | | |
|---|---|---|---|---|---|---|---|
| Of 1 case at the age of | 9 years, | | no deaths. | | | | |
| " 41 " " " " | 11-20 | " | " | " | | | |
| " 43 " " " " | 21-30 | " | 4 | " | or, | 9.30 | p.c. |
| " 27 " " " " | 31-40 | " | 1 | " | " | 3.70 | " |
| " 15 " " " " | 41-50 | " | 2 | " | " | 13.33 | " |
| " 17 " " " " | 51-60 | " | 8 | " | " | 47.06 | " |
| " 9 " " " " | 61-70 | " | 5 | " | " | 55.55 | " |
| " 6 " " " " | 71-80 | " | 5 | " | " | 83.33 | " |
| " 1 " " " " | -94 | " | no | " | | | |

With the exception of the cases between thirty-one and forty years of age, whose small mortality may be an accidental occurrence, age steadily increases the tendency to a fatal termination, which is even more strikingly shown by this compilation:

| | | | | | |
|---|---|---|---|---|---|
| Of 127 patients | below 50 years, | 7 deaths, | or, | 5.5 | p.c. |
| " 33 " | above 50 " | 18 " | " | 54.5 | " |

(*To be continued.*)

